# General Habit Propensity Relates to the Sensation Seeking Subdomain of Impulsivity But Not Obesity

**DOI:** 10.3389/fnbeh.2016.00213

**Published:** 2016-11-08

**Authors:** Anja Dietrich, Sanne de Wit, Annette Horstmann

**Affiliations:** ^1^Department of Neurology, Max Planck Institute for Human Cognitive and Brain SciencesLeipzig, Germany; ^2^Department of Clinical Psychology, University of AmsterdamAmsterdam, Netherlands; ^3^IFB Adiposity Diseases, Leipzig University Medical CenterLeipzig, Germany; ^4^Collaborative Research Center A052A5, Leipzig University Medical CenterLeipzig, Germany

**Keywords:** obesity, BMI, impulsivity, sensation seeking, habitual, goal-directed, instrumental discrimination

## Abstract

According to dual-system theory, instrumental learning and performance depend on the balance between goal-directed and habitual action control. Overreliance on habits has been argued to characterize clinical conditions such as drug addiction or obsessive-compulsive disorder as well as obesity and excessive impulsivity. A tendency toward habitual action control in obesity has already been indicated in the food domain. However, impairments might not be restricted to eating behavior. This has been suggested by domain-general obesity-associated disturbances in executive function as well as alterations in corticostriatal circuits underlying the goal-directed and habitual systems. In this study we examined the balance of goal-directed and habitual action control in a sample of normal-weight, overweight, and obese participants (*n* = 105) using the *slips-of-action test* in a non-food context. We tested for continuous or group-based associations between *body weight status (BMI)* and the *devaluation sensitivity index (DSI)*, a parameter representing the balance of the goal-directed and habitual systems in action control. As personality differences in the domain of impulsivity might affect this relationship, we also examined whether the interaction between BMI and self-reported impulsivity, based on the *UPPS Impulsive Behavior Scale*, was related to the DSI. In addition to that, we tested for direct, i.e., weight status independent, relationships between UPPS subdomains of impulsivity and the DSI. We failed to find evidence for a relationship between weight status and sensitivity to devaluation as indexed by the DSI. However, independent of weight status, we observed lower sensitivity to devaluation in *sensation seekers*, a subtype of impulsivity. To conclude, behavioral flexibility in the sense of disturbances in the balance between the habitual and goal-directed systems seems to be unaffected by weight status in a non-food context. Consequently, stimuli and behavior might not be generally excessively linked in overweight or obesity. However, according to ceiling effects we cannot rule out subtle effects the paradigm was not able to disentangle. Further, future studies are needed to clarify the role of specific subtypes of obesity (e.g., food addiction). The indicated habit propensity in *sensation seekers* may account for previous reports of weak avoidance behavior and risky decision making.

## Introduction

According to dual-system accounts, the flexibility of instrumental action is in part determined by the balance between goal-directed and habitual action control systems (for associative accounts, see e.g., Dickinson, 1985; de Wit and Dickinson, 2009). The goal-directed system encodes associations between actions [responses (R)] and their consequences [outcomes (O)] which are indicated by environmental cues [stimuli (S)]. Habitual action, conversely, is driven by S→R associations only, without consideration of outcome values (de Wit and Dickinson, 2009). The habit system is thought to dominate with excessive training, as it is more efficient than the goal-directed system in a stable environment, though at the expense of flexibility (Dickinson, 1985; de Wit and Dickinson, 2009). Overreliance on habits supposedly characterize clinical conditions that involve impulsive and compulsive behavior, as indicated by instrumental decision-making tasks (i.e., selective outcome devaluation procedures or model-free/model-based reinforcement learning paradigms) that assess the ability to rapidly change behavior to changes in outcome value. Such conditions include obsessive-compulsive disorder (Gillan et al., 2011, 2014), drug addiction (e.g., Everitt and Robbins, 2005; Everitt et al., 2008; Redish et al., 2008; Sjoerds et al., 2013; Voon et al., 2015), and obesity (Volkow and Wise, 2005; Horstmann et al., 2015a). Further, predominance of habitual control has been argued to contribute to individual differences in compulsive tendencies (Snorrason et al., 2016) and impulsivity (Everitt et al., 2008; Hogarth et al., 2012) in non-clinical samples, indicated by outcome devaluation tasks.

Maladaptive habitual behavior is a frequently discussed issue in obesity research (Volkow and Wise, 2005; Corbit, 2016). In a recent study we showed that body weight status was positively related to heightened habit-like behavior in the food domain (Horstmann et al., 2015a). However, behavior might be generally less flexible and goal-directed in obesity. This is indicated by lower performance in tasks assessing domain-general executive function in healthy young participants (reviewed in Vainik et al., 2013), especially inhibitory control (Nederkoorn et al., 2006; Gunstad et al., 2007) and decision making (Pignatti et al., 2006; Weller et al., 2008; Horstmann et al., 2011; Simmank et al., 2015). For instance, there is evidence for obesity-associated impulsive decision making using the delay-discounting task. In this task participants with obesity showed preference of small immediately available monetary rewards over larger delayed ones (Weller et al., 2008; Simmank et al., 2015). Additionally, self-reported impulsivity has been shown to be increased in obesity (e.g., Rydén et al., 2003; Braet et al., 2007; Guerrieri et al., 2008; Mobbs et al., 2010). Impulsivity is a complex trait, including the inability to withhold responses in the face of negative consequences, the preference for small immediate rewards vs. larger delayed ones, acting without forethought, as well as a tendency for sensation seeking and increased disposition to engage in risky behaviors (reviewed in Bari and Robbins, 2013). It is a relevant trait which needs consideration in the investigation of instrumental action control, and may moderate the potential relationship between weight status and the domain-general balance between goal-directed and habitual control. Impulsivity has been proposed to be accompanied by an overreliance on habits at the expense of goal-directed behavioral control (Everitt et al., 2008; Hogarth et al., 2012). Hogarth et al. (2012) assessed individual differences between self-reported impulsivity and goal-directed behavioral control using a food-dependent devaluation procedure. After instrumental training of response-outcome contingencies a chocolate snack was devalued, induced by specific satiety. Testing responding for the devalued outcome (chocolate snack) in extinction revealed impaired goal-directed behavioral control with respect to the aspect of motor impulsivity (i.e., action without thought).

Although previous research suggest behavioral inflexibility already in healthy obese individuals, abnormalities in habit formation might be especially pronounced in certain subtypes. For example, similar to drug addiction the obesity- and impulsivity-associated psychopathology of food addiction (Gearhardt et al., 2009; Meule and Gearhardt, 2014) may be related to impaired goal-directed action control in favor of habitual behavior. Apart from that, behavioral autonomy may also differ between mild and morbid obesity. According to a recently proposed model for dopamine and the severity of obesity (Horstmann et al., 2015b), individuals with overweight and mild obesity may be characterized by a low dopaminergic tone and enhanced behavioral flexibility, whereas morbid obesity might be characterized by a diminished dopaminergic tone and behavioral rigidity.

Both impulsivity and obesity are associated with abnormalities in corticostriatal and corticolimbic pathways [(impulsivity: e.g., Jentsch and Taylor, 1999; Volkow and Fowler, 2000; Cardinal et al., 2001; McClure et al., 2004; Brown et al., 2006; Dalley et al., 2008; Forbes et al., 2009; Davis et al., 2013), (obesity: e.g., Stoeckel et al., 2009; Berridge et al., 2010; Horstmann et al., 2011; Volkow et al., 2011; Dimitropoulos et al., 2012; Nummenmaa et al., 2012; Garcia-Garcia et al., 2013; Kullmann et al., 2014)] underlying goal-directed and habitual behavior (Balleine and O’Doherty, 2010). More specifically, goal-directed control has been associated with function and structure of the ventromedial prefrontal cortex, orbitofrontal cortex, cingulate cortices as well as caudate nucleus (Hampton and O’doherty, 2007; Valentin et al., 2007; Tanaka et al., 2008; de Wit et al., 2009, 2012b; Gläscher et al., 2009; Tricomi et al., 2009). The posterior putamen, on the other hand, seems to be involved in habit learning (Tricomi et al., 2009; de Wit et al., 2012b). Links between dysregulated neurotransmitter systems, involved in modulating the balance of goal-directed vs. habitual action control (de Wit et al., 2012a; Worbe et al., 2015), and impulsivity (Dolan et al., 2002; King et al., 2003; Dalley et al., 2008; Forbes et al., 2009; Buckholtz et al., 2010) or obesity (Wang et al., 2001; de Weijer et al., 2011; Eisenstein et al., 2013; Guo et al., 2014; Horstmann et al., 2015b) support the possibility of a general impairment in goal-directed behavior.

We hypothesized body weight status [as measured by the *body mass index* (BMI)], food addiction as well as impulsivity to be characterized by a disruption in the balance between goal-directed and habitual action control, resulting in overreliance on habits. Further, we expected impulsivity to moderate the relationship between BMI and measures of goal-directed and habitual behavioral control. To investigate these hypotheses, a sample of young and healthy normal-weight, overweight and obese volunteers were assessed on different aspects of self-reported impulsivity (Whiteside and Lynam, 2001) and food addiction (Gearhardt et al., 2009; Meule and Gearhardt, 2014). In addition to these critical analyses we explored the influence of gender on the balance between the goal-directed and the habitual system. This was suggested by previous research indicating gender differences in the sensitivity to reinforcement. Gonadal hormones and their effects on the dopaminergic system supposedly account for these differences (Sofuoglu et al., 1999; Kaasinen et al., 2001; Evans et al., 2002; Lynch et al., 2002; Carroll et al., 2004). Importantly, a previous study using the same instrumental paradigm as applied here, showed an effect of dopamine depletion on instrumental decision-making in women only (de Wit et al., 2012a).

Individual differences in action control were measured using an established instrumental learning task (de Wit et al., 2007, 2012b; Gillan et al., 2011; Worbe et al., 2015; Delorme et al., 2016). In this task, participants were trained to perform specific responses which were indicated by certain associated stimuli, to obtain rewarding outcomes. In the critical ‘slips-of-action’ test phase, some of the outcomes were devalued, allowing evaluation of participants’ ability to adapt responding based on the current goal or outcome value, as opposed to relying on inflexible stimulus-response habits. We restricted the experimental procedure to the standard discrimination type of the original ‘slips-of-action’ test phase, as this discrimination type has been shown to be sufficiently sensitive to evaluate the balance between habitual and goal-directed action control in clinical and pre-clinical pathological conditions (Gillan et al., 2011; Delorme et al., 2016; Snorrason et al., 2016) and after pharmacological manipulations affecting dopamine and serotonin neurotransmission (de Wit et al., 2012a; Delorme et al., 2016).

## Materials and Methods

### Participants

We investigated 105 (60 females) healthy normal-weight (BMI >= 19 < 25), overweight (BMI >= 25 < 30) and obese volunteers (BMI > 30) recruited from the subject database of the Max Planck Institute for Human Cognitive and Brain Sciences in Leipzig. Volunteers were healthy non-smokers without indication for major depression (Beck’s Depression Inventory, cut-off value 18, Beck et al., 1961). Prior to participation in the study participants gave written informed consent in accordance with the Declaration of Helsinki and the requirements of the local ethics committee of the University of Leipzig. Please see **Table 1** (grouping by weight status) and **Table 2** (grouping by gender) for details on the samples’ descriptive statistics (Supplementary Table S1 of the Supplementary Material shows correlations between the investigated variables).

**Table 1:** Descriptive statistics of the sample by *weight status.*

	Normal-weight (*n* = 36)	Overweight (*n* = 35)	Obese (*n* = 34)	Kruskal–Wallis *H*	*p*
BMI (kg/m^2^)	20.26 / **21.72** / 23.35	21.18 / **26.77** / 27.78	31.18 / **33.13** / 36.43	92.440	**<0.001**
YFAS symptoms	0.0 / **1.0** / 1.0	0.0 / **1.0** / 2.0	1.0 / **2.0** / 3.0	15.727	**<0.001**
Age	24.3 / **27.0** / 29.8	24.0 / **25.0** / 28.0	25.0 / **27.0** / 29.3	2.011	0.366
UPPS Urgency	23.25 / **26.0** / 30.0	22.0 / **27.0** / 32.0	23.5 / **27.0** / 31.5	0.165	0.921
UPPS (Lack of) Premeditation	19.75 / **22.0** / 24.0	18.0 / **23.0** / 25.0	21.0 / **23.0** / 24.0	0.015	0.992
UPPS (Lack of) Perseverance	15.25 / **19.0** / 22.75	16.0 / **21.0** / 25.0	16.0 / **19.5** / 23.75	1.584	0.453
UPPS Sensation Seeking	25.25 / **32.0** / 37.75	28.0 / **33.0** / 35.0	25.5 / **31.5** / 36.25	0.121	0.942
IQ	111.75 / **125.25** / 130.0	114.0 / **123.5** / 127.0	110.25 / **119.0** / 130.0	0.698	0.705
VPA score	10.0 / **12.5** / 15.0	11.0 / **13.0** / 14.0	9.75 / **13.0** / 15.0	0.130	0.937
BDI	1.25 / **3.0** / 6.0	3.0 / **5.0** / 9.25	1.0 / **3.5** / 7.25	4.312	0.116

***n* females / males**				**Chi^2^**	***p***

Gender	22 / 14	19 / 16	19 / 15	χ^2^: 2.143	0.143

*Including body mass index (BMI), number of symptoms on the Yale Food Addiction Scale (Gearhardt et al., 2009; Meule et al., 2012), age, impulsivity scores based on the UPPS Impulsive Behavior Scale (Whiteside and Lynam, 2001), IQ (Wiener Matritzen-Test, Formann, 1979), visual short-term memory based on the Visual Paired Associates Test (VPA) of the Wechsler Memory Scale (Wechsler, 1987, 2006), depression symptoms (Beck’s Depression Inventory, Beck et al., 1961), and gender. Indicated are 1st Quartile, Median (bold), and 3rd Quartile as well as group differences based on the Kruskal–Wallis test or χ^2^-Test (gender).*

**Table 2:** Descriptive statistics of the sample by *gender.*

	Females (*n* = 60)	Males (*n* = 45)	Mann–Whitney *U*	*p*
BMI (kg/m^2^)	22.07 / **26.55** / 32.10	23.40 / **27.47 /** 31.13	1265.5	0.584
YFAS symptoms	1.0 / **1.0** / 2.0	0.0 / **0.0** / 1.0	548.0	**<0.001**
Age	23.0 / **25.0** / 28.0	25.0 / **27.0** / 30.0	951.0	**0.009**
UPPS Urgency	23.0 / **28.0** / 33.0	22.25 / **26.0** / 29.0	1068.5	0.097
UPPS (Lack of) Premeditation	18.25 / **22.5** / 24.0	21.0 / **23.0** / 24.5	1193.0	0.307
UPPS (Lack of) Perseverance	16.0 / **20.0** / 22.75	16.5 / **20.0** / 24.0	1252.0	0.525
UPPS Sensation Seeking	24.25 / **30.50** / 35.0	30.0 / **34.0** / 37.5	973.5	**0.015**
IQ	111.75 /**123.5** / 130.0	112.5 /**123.5** /130.0	1248.0	0.507
VPA score	11.0 / **13.0** / 15.0	10.0 / **12.0** / 15.0	1136.5	0.165
BDI score	2.0 / **4.0** / 7.0	1.0 / **4.0** / 8.0	1284.5	0.814

*Including BMI, number of symptoms on the Yale Food Addiction Scale (Gearhardt et al., 2009; Meule et al., 2012), age, impulsivity scores based on the UPPS Impulsive Behavior Scale (Whiteside and Lynam, 2001), IQ (Wiener Matritzen-Test, Formann, 1979), visual short-term memory based on the VPA of the Wechsler Memory Scale (Wechsler, 1987, 2006), and depression symptoms (Beck’s Depression Inventory, Beck et al., 1961). Indicated are 1st Quartile, Median (bold), and 3rd Quartile as well as gender differences based on the Mann-Whitney *U* test.*

### Questionnaires

#### UPPS Impulsive Behavior Scale (Whiteside and Lynam, 2001; German: Schmidt et al., 2008)

The UPPS Impulsive Behavior Scale consists of 45 items that are rated on a 4-point Likert scale ranging from 1 (*strongly agree*) to 4 (*strongly disagree*). It contains four subscales corresponding to four facets of impulsivity: *Urgency, (Lack of) Premeditation, (Lack of) Perseverance*, and *Sensation Seeking*. The 12-item *Urgency* scale “refers to the tendency to experience strong impulses, frequently under conditions of negative affect” (Whiteside and Lynam, 2001). *(Lack of) Premeditation*, an 11-item scale, “refers to the tendency to think and reflect on the consequences of an act before engaging in that act” (Whiteside and Lynam, 2001). *(Lack of) Perseverance*, a 10-item scale, “refers to an individual’s ability to remain focused on a task that may be boring or difficult” (Whiteside and Lynam, 2001). The 12-item *Sensation Seeking* scale “incorporates two aspects: (1) a tendency to enjoy and pursue activities that are exciting and (2) an openness to trying new experiences that may or may not be dangerous” (Whiteside and Lynam, 2001). The German adaptation robustly confirms the four-factor dimensionality of the original version with very good internal consistency of the four subscales (Cronbach’s α range: 0.80–0.85; Schmidt et al., 2008). Please see **Table 1** (grouping by weight status) and **Table 2** (grouping by gender) for sample characteristics regarding the UPPS subdomains.

#### Yale Food Addiction Scale (Gearhardt et al., 2009; German: Meule et al., 2012)

The Yale Food Addiction Scale is a standardized instrument to identify people with distinctive symptoms indicative of addiction to certain foods. It is a 25-item self-report questionnaire assessing seven food addiction symptoms [based on the seven substance dependence criteria of the *Diagnostic and Statistical Manual of Mental Disorders* (DSM-IV); DSM-IV-TR, 2000] as well as clinically significant impairment or distress. Individuals rate their eating behavior during the last 12 months, referring specifically to high fat and high sugar foods. Ratings comprise a combination of dichotomous and frequency scoring. A symptom is met if at least one question of that criterion is scored as one. A continuous symptom count (range: 0–7) is calculated, adding symptoms which have been met. Additionally, a dichotomous score is calculated to “diagnose” food addiction. Food addiction is diagnosed if at least three symptoms and the criterion of a clinically significant impairment or distress is met. The German version replicates the original one-factorial structure with adequate internal consistency (Cronbach’s α = 0.81–0.83; Meule and Kübler, 2012; Meule et al., 2012). Sample characteristics on the YFAS are shown in **Table 1** (grouping by weight status) and **Table 2** (grouping by gender).

### Higher-Order Cognitive Measures: IQ and Visual Short-Term Memory

The goal-directed system has been indicated to depend on cognitive capacities (Eppinger et al., 2013; Otto et al., 2013; Schad et al., 2014). Therefore measures of higher-order cognitive function (i.e., IQ and visual short-term memory) were considered as control variables. Non-verbal IQ was determined by the Wiener Matritzen-Test (WMT, score range: 0–24 which translates into an IQ range of 60.5–136.5; Formann, 1979). To estimate visual short-term memory, we used a computerized version of the Visual Paired Associates Test (VPA, score range: 0–18), a subtest of the Wechsler Memory Scale (Wechsler, 1987, 2006). Please see **Table 1** (grouping by weight status) and **Table 2** (grouping by gender) for sample characteristics on IQ and visual short-term memory.

### Experimental Paradigm

We applied a simplified version of an established instrumental learning paradigm (e.g., de Wit et al., 2007, 2012b; Gillan et al., 2011). The following description of the paradigm relates to the main task characteristics. Please see Worbe et al. (2015) for further details on the simplified version. The task was programmed in Visual Basic 6.0. In contrast to the original version, participants were presented with animal icons instead of fruit pictures, as we were interested in instrumental performance in a non-food context. The paradigm consisted of four stages: discrimination training phase, outcome-devaluation test, slips-of-action and baseline tests, and questionnaires on contingency knowledge.

#### Stage 1: Discrimination Training Phase

**FIGURE 1:**
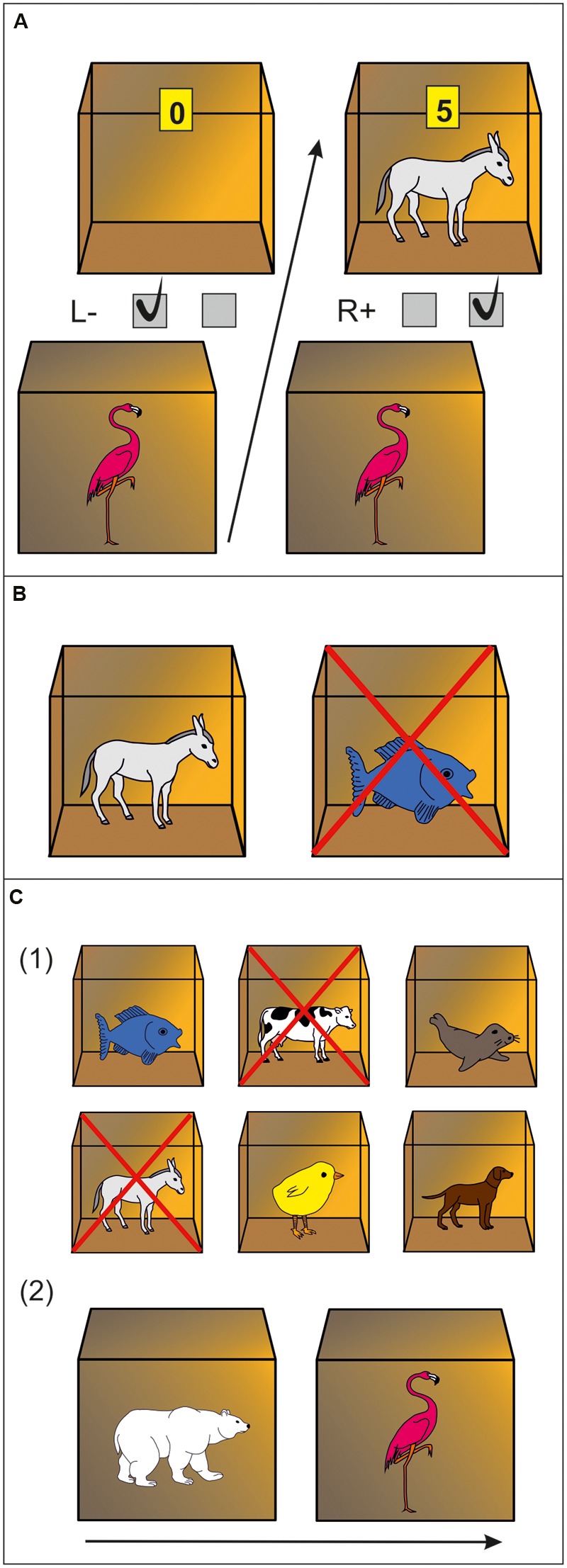
**Experimental paradigm. (A)** Discrimination training phase. In this example, a flamingo stimulus outside of a box indicates that pressing the right key will be rewarded with a donkey and points inside of the box. Pressing the left key will not be rewarded (empty box is revealed). **(B)** Outcome-devaluation test. In this example, two open boxes are presented with a donkey and fish inside. The cross superimposed on the fish signals this outcome is no longer worth any points. The accurate response in this example would be pressing the right key (which yielded the still-valuable donkey outcome during the training phase). **(C)** Slips-of-action test. (1) Participants are first presented with the six outcomes. In this example, donkey and cow are superimposed with a cross, indicating that the response leading to these outcomes will now result in subtraction of points (devaluation). The other animal outcomes are still valuable. (2) Afterwards, a rapid succession of animal stimuli are presented outside of boxes. Participants are instructed to press the correct keys if a stimulus indicates the availability of a still-valuable outcome inside the box (“Go,” example: polar bear stimulus signaling fish outcome), but withhold responding if the outcome inside the box has been devalued (“No-Go,” example: flamingo stimulus signaling donkey outcome).

Twelve animal icons either functioned as stimulus or outcome, resulting in six associative pairs. Participants had to memorize these pairs and the respective associated key press. In detail, on each trial an animal icon [stimulus (S)] outside of a box signaled left or right key press [response (R)]. If the response was correct, an animal icon appeared inside of the box and points were earned [outcome (O)]. If the response was incorrect, the box remained empty and no points were earned. The number of points that could be earned for a correct key press depended on response latency (de Wit et al., 2007). For example, by trial and error participants should learn that an flamingo outside of the box signaled pressing the right key which would be rewarded with a donkey inside and points. Pressing the left key, on the other hand, would result in an empty box and no points (**Figure 1A**). Participants were informed that they would receive one euro-cent for each collected point. Assignment of the animal pictures to the stimulus vs. outcome sets was counterbalanced. Every stimulus was presented twice in each of eight blocks, resulting in 96 trials altogether. Picture presentation was randomized within each block.

#### Stage 2: Outcome-Devaluation Test on Response-Outcome Knowledge

This stage was to test if participants learned R→O contingencies. On every trial, two open boxes with animals inside were shown, one that had previously been associated with a left key press and the other one with a right key press. One of these animals (i.e., outcomes) was no longer rewarded, indicated by a cross which was superimposed on this animal (**Figure 1B**). Participants were instructed to press the key which was previously rewarded by the still-valuable outcome. Feedback was no longer provided, although the participants were told, that correct key presses would still earn them points. Every outcome was devalued twice, resulting in 36 trials altogether.

#### Stage 3: Slips-of-Action Test

This test was designed to assess the balance between goal-directed (S→O→R) vs. habitual (S→R) behavior. Each of nine blocks started with a 5 s presentation of all six animal outcomes, with two of them being devalued, indicated by a cross superimposed [**Figure 1C** (1)]. Afterwards, participants were presented a series of closed boxes with animal stimuli outside for 1 s each. They were instructed to no longer open boxes (i.e., withhold response) if the stimulus outside was linked to a devalued outcome inside, but press the appropriate key before a stimulus disappeared which signaled a still-valuable outcome [**Figure 1C** (2)]. Strong direct S→R associations should trigger responses regardless of the current value of the outcome, resulting in “slips of action” toward devalued outcomes. Good task performance is achieved if participants are able to base responding on the current value of the signaled outcome using S→O→R associations. Altogether the test consisted of 108 trials (72 still-valuable and 36 devalued). Participants did not receive feedback but were told that they would still earn points for correct responses for still-valuable outcomes but lose points for responses for devalued outcomes. To assess the balance between goal-directed and habitual control, a devaluation sensitivity index (DSI) was computed by subtracting the percentage of responses for devalued outcomes from that for still-valuable outcomes (Gillan et al., 2011; de Wit et al., 2012a; Snorrason et al., 2016). This index represents the relative involvement of the habit vs. goal-directed system in action control, i.e., high scores indicate strong goal-directed responding, whereas low scores suggest a habit propensity.

In addition, a baseline test was included. It was identical to the slips-of-action test, except that stimuli were devalued instead of outcomes, i.e., it does not require S→O→R knowledge. Therefore, this test controls for goal-directed behavior but still depends on S→R knowledge. Participants were shown the six stimulus animals with two of them devalued. They were instructed to withhold response for the two devalued ones. A baseline DSI was calculated by subtracting responding for devalued stimuli from responding for still-valuable stimuli. The order of the two tests was counterbalanced.

#### Stage 4: Questionnaires of Contingency Knowledge

Paper-and-pencil questionnaires were used to measure explicit knowledge of the contingencies. Participants had to indicate: which response was correct for each of the six stimuli (S→R questionnaire), which outcome was associated with which stimulus (S→O questionnaire), and which response was awarded with which of the six outcomes (R→O questionnaire). In addition to that, they indicated on visual analog scales how certain they were about their answers. Questionnaire order was counterbalanced.

### Statistical Analysis

Outcome measures were generally non-normally distributed (Kolmogorov–Smirnov test, see Supplementary Table S2; Supplementary Figure S1). Therefore, non-parametric tests were used for statistical analysis. The Friedman test was applied to test for performance differences between instrumental learning blocks. First we tested for continuous relationships between weight status or aspects of impulsivity and instrumental learning (discrimination training), performance during the critical test phase (outcome-devaluation test, slips-of-action test) and explicit knowledge of contingencies (questionnaires). For this, spearman correlations were conducted to inspect associations between BMI or aspects of impulsivity (UPPS scales) and total accuracy during the discrimination training, accuracy on the outcome-devaluation test, the DSI of the slips-of-action and baseline tests and certainty of explicit contingency knowledge. In addition to BMI and impulsivity, control variables (IQ and VPA score) were tested for correlations with the mentioned outcome measures. If control variables correlated with outcome measures, they were controlled for in the corresponding correlation analyses of BMI and the UPPS impulsivity domains using partial rank correlation. Moderation analysis on ranks were performed to examine whether the level of impulsivity moderated the relationship between BMI and accuracy on the outcome-devaluation test or the DSI of the slips-of-action and baseline tests. In addition to these continuous analyses we compared normal-weight, overweight and obese participants groupwise regarding the above-mentioned outcome measures. Differences were evaluated using rank analysis of covariance, including, depending on significant associations with the outcome measures, VPA and/or IQ as covariates. According to the sample’s low variance, food addiction (Gearhardt et al., 2009; Meule et al., 2012) was not explicitly investigated regarding associations with task performance as analyses would not yield meaningful results (see **Tables 1** and **2**, only seven participants identified as food-addicted). To rule out gender effects in learning of the associations and performance in the critical test phase the following analyses were additionally conducted: Gender differences in learning during the discrimination training phase were evaluated using an 8 (block) × 2 (gender) ANOVA on ranks, including – due to gender differences in these variables (see **Table 2**) – age and UPPS *Sensation Seeking* as covariates. Rank analysis of covariance was conducted to test for gender differences in test performance, i.e., accuracy on the outcome-devaluation test and the DSI of the slips-of-action and baseline tests (covariates: age, UPPS *Sensation Seeking*). SPSS version 20.0 (IBM Corporation, Somers, NY, USA) was used for data analysis.

## Results

### Discrimination Training Phase

**FIGURE 2:**
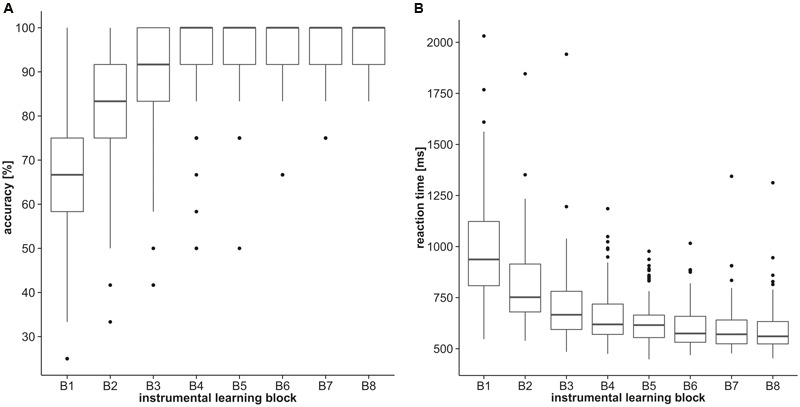
**Discrimination training performance.** Boxplots represent **(A)** accuracy (percentages of correct responses) and **(B)** reaction times of the eight discrimination training blocks.

Participants rapidly learned the instrumental discriminations. With progression of the training phase accuracy (percentages of correct responses) increased steadily [first block = *Mdn*: 67%, interquartile range (*IQR*): 58–75%; last block = *Mdn*: 100%, *IQR*: 91–100%; **Figure 2A**] and reaction times decreased (first block = *Mdn*: 937.0 ms, *IQR*: 808.5–1124.0 ms; last block = *Mdn*: 560.5 ms, *IQR*: 523.5–633.0 ms; **Figure 2B**). Performance during the eight blocks significantly differed from each other (Friedman Test, χ^2^ = 391.02, *p* < 0.001), emphasizing the descriptively outlined learning effect. Control measures affected learning performance. Total accuracy during the training phase (averaged over all blocks) positively correlated with estimates of IQ (ρ = 0.304, *p* = 0.002) and visual short-term memory (ρ = 0.282, *p* = 0.004). Controlling for these variables, total accuracy during the training phase was not continuously associated with BMI (ρ = 0.075, *p* = 0.451) nor any assessed measure of impulsivity (UPPS scales: ρ <= 0.116, *p* >= 0.200), indicated by partial rank correlations. A BMI-based group analysis [8 (block) by 3 (group) ANCOVA on ranks, covariates: VPA, IQ] confirmed the learning effect [main effect of block: *F*(7,700) = 2.088, *p* = 0.043], but corresponding to the aforementioned correlation analysis BMI was not indicated to significantly affect performance during training [main effect of group: *F*(2,100) = 0.50, *p* = 0.608; block^∗^group: *F*(14,700) = 0.803, *p* = 0.666]. Gender did not affect instrumental learning during the training indicated by repeated measures 8 (block) by 2 (gender) ANCOVA on ranks (covariates: UPPS *Sensation Seeking*, age) yielding no significant main effects for block [*F*(7,101) = 0.868, *p* = 0.531] or gender [*F*(1,101) = 0.890, *p* = 0.348] nor a significant block^∗^gender interaction [*F*(7,707) = 1.303, *p* = 0.246].

### Outcome-Devaluation Test

Performance was generally very good and in fact near ceiling (**Table 3**; see Supplementary Figure S1 of the Supplementary Material for a histogram and Supplementary Table S3 for reaction times). We first tested whether control measures were associated with accuracy (percentage of correct responses) in this test. IQ was unrelated, but we did find a significant positive relationship with visual short-term memory: The higher the VPA score was, the more accurately participants responded for the still-valuable outcomes (ρ = 0.277, *p* = 0.004). Controlling for visual short-term memory, we did not find a continuous relationship between accuracy during the devaluation test and BMI as assessed by partial rank correlation (ρ = -0.09, *p* = 0.926). Also group analysis (rank analysis of covariance, covariate: VPA) did not suggest differences between normal-weight, overweight and obese participants regarding accuracy of responding for still-valuable outcomes [*F*(2,104) = 0.201, *p* = 0.818]. Interestingly, UPPS *Sensation Seeking* was negatively associated with accuracy during this test: The higher participants scored in this domain of impulsivity the less accurate their choices were (ρ = -0.197, *p* = 0.045). This correlation remained significant after controlling for the other UPPS scales using partial rank correlation (ρ = -0.217, *p* = 0.030). Spearman’s correlation analyses did not yield significant relationships between test accuracy and any other UPPS impulsivity domain (ρ <= 0.123, *p* >= 0.223). A gender difference in accuracy after devaluation, as determined by rank analysis of covariance, was not observed [*F*(1,103) = 0.733, *p* = 0.394; covariates: age, UPPS *Sensation Seeking*].

**Table 3:** Task performance in the slips-of-action, baseline, and outcome-devaluation tests (1st Quartile, Median, and 3rd Quartile).

	Slips-of-action test			Baseline test			Outcome-devaluation test
interquartile range	Devalued (%)	Valuable (%)	DSI	Devalued (%)	Valuable (%)	DSI	Accuracy (%)
1st Quartile	5.56	74.31	54.86	4.17	84.03	70.14	88.89
Median	11.11	87.50	76.39	8.33	93.06	83.33	94.44
3rd Quartile	20.83	94.44	88.19	13.89	97.22	89.58	100.00

*Included are the percentages of responding for devalued and still-valuable outcomes as well as the corresponding devaluation sensitivity index (DSI) in the slips-of-action test; the percentages of responding for devalued and still-valuable stimuli including the corresponding DSI in the baseline test, and accuracy in the outcome-devaluation test.*

### Slips-of-Action and Baseline Tests

Performance in both tests was very good. There were just few responses for cues indicating devalued outcomes or stimuli and high responding for cues indicating still-valuable outcomes or stimuli (**Table 3**; see Supplementary Figure S1 of the Supplementary Material for histograms and Supplementary Table S3 for reaction times). Descriptive data on the DSI of the slips-of-action test indicated a general trend toward outcome-based responding (**Table 3**).

**FIGURE 3:**
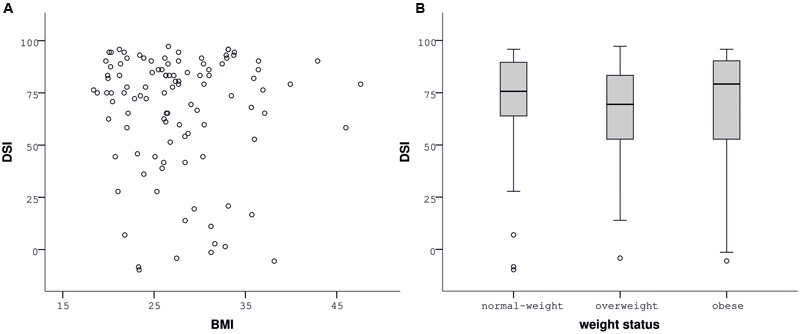
**Performance in the slips-of-action test depending on body mass index (BMI).** The devaluation sensitivity index (DSI) did not correlate with BMI **(A)** or differ between normal-weight, overweight, and obese participants **(B)**.

**FIGURE 4:**
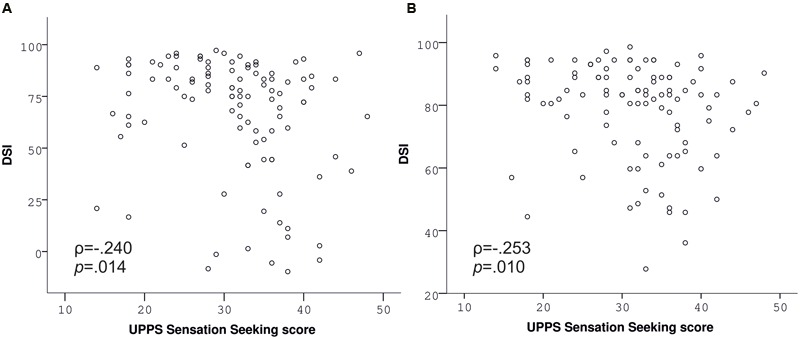
**Performance in the slips-of-action** **(A)** and baseline test **(B)** depending on UPPS *Sensation Seeking*. UPPS *Sensation Seeking* is negatively associated with devaluation sensitivity indices (DSI) of both tests (Spearman correlations).

Contrary to our hypothesis, BMI was unrelated to the DSI of the slips-of-action (ρ = -0.029, *p* = 0.770, **Figure 3A**) as well as the baseline test (ρ = 0.033, *p* = 0.738), indicated by correlation analyses. Correspondingly, group analyses did not indicate differences in the sensitivity to devaluation between normal-weight, overweight, and obese participants [slips-of-action test: *F*(2,104) = 0.577, *p* = 0.563, **Figure 3B**; baseline test: *F*(2,104) = 0.079, *p* = 0.924]. In line with our hypothesis, impulsivity, i.e., UPPS *Sensation Seeking*, was negatively associated with the DSI of the slips-of-action (ρ = -0.240, *p* = 0.014; **Figure 4A**) and baseline test (ρ = -0.253, *p* = 0.01; **Figure 4B**), meaning that the higher participants scored in *Sensation Seeking* the less they were sensitive to devaluation of both outcomes and stimuli. Correlation between UPPS *Sensation Seeking* and the DSI remained significant after controlling for the other UPPS domains of impulsivity (slips-of-action test: ρ = -0.270, *p* = 0.007; baseline test: ρ = -0.326, *p* = 0.001). No other UPPS scale was related to devaluation sensitivity (ρ <= 0.094, *p* >= 0.359). We further assessed whether impulsivity (i.e., UPPS *Sensation Seeking*) moderated the relationship between BMI and task performance in the slips-of-action test, but did not find evidence for a significant interaction with the DSI [*F*(1,101) = 0.567, *p* = 0.453]. As control measures were positively correlated with the DSI of the slips-of-action (IQ: ρ = 0.370, *p* < 0.001; VPA: ρ = 0.418, *p* < 0.001) and baseline test (IQ: ρ = 0.274, *p* = 0.005; VPA: ρ = 0.394, *p* < 0.001), the previously mentioned correlation and group analyses were controlled for IQ and VPA score. Gender did not affect the DSI of the slips-of-action test [*F*(1,103) = 2.947, *p* = 0.089, covariates: age, UPPS *Sensation Seeking*] or baseline test [*F*(1,103) = 3.195, *p* = 0.077, covariates: age, UPPS *Sensation Seeking*], as determined by rank analysis of covariance.

### Questionnaires of Contingency Knowledge

Variance of questionnaire measures was extremely low, with a majority of participants accomplishing 100% accuracy and full certainty regarding all kinds of associations (see **Table 4**; Supplementary Figure S2 of the Supplementary Material for histograms). According to strong ceiling effects, we decided not to perform correlation analyses with respect to the accuracy measures (100% accuracy: S→R = 98%, R→O = 79%, SO = 80% of participants) and certainty of S→R assignments (77% of participants with maximum certainty), as they would not give meaningful results. According to less pronounced ceiling, certainty of R→O (51% of participants reaching maximum certainty) and S→O associations (42% of participants reaching maximum certainty) were inspected for correlations with the variables of interest, excluding participants reaching maximum certainty beforehand. Both control measures were positively associated with R→O (IQ: ρ = 0.389, *p* = 0.004; VPA: ρ = 0.381, *p* = 0.004; *n* = 54) and S→O certainties (IQ: ρ = 0.320, *p* = 0.011; VPA: ρ = 0.414, *p* = 0.001; *n* = 63) and controlled for in the following analyses. BMI and UPPS *Sensation Seeking* did not correlate with the certainty of R→O (BMI: ρ = 0.066, *p* = 0.634; UPPS *Sensation Seeking*: ρ = -0.162, *p* = 0.242; *n* = 54) and S→O (BMI: ρ = 0.119, *p* = 0.352; UPPS *Sensation Seeking*: ρ = -0.101, *p* = 0.432; *n* = 63) knowledge. In addition to that, the comparison of normal-weight, overweight, and obese participants did not indicate group differences in the certainty of R→O [*F*(2,53) = 1,294, *p* = 0.283] and S→O [*F*(2,62) = 0.561, *p* = 0.573] knowledge.

**Table 4:** Explicit knowledge (1st Quartile, Median, and 3rd Quartile).

Interquartile range	S→R accuracy (%)	S→R certainty	R→O accuracy (%)	R→O certainty	S→O accuracy (%)	S→O certainty
1^st^ Quartile	100.00	9.15	100.00	8.51	100.00	7.44
Median	100.00	10.00	100.00	9.90	100.00	9.77
3^rd^ Quartile	100.00	10.00	100.00	10.00	100.00	10.00

*Accuracy (percentages of correct answers) and certainty of S→R, R→O, and S→O assignments (certainty_max_ = 10, as measured by visual analog scales).*

## Discussion

The present study addressed the two main questions: (1) Whether there is a domain-general habit propensity in obesity; (2) And whether aspects of impulsivity influence the balance between goal-directed and habitual action control. Regarding excess body weight, we did not find evidence for heightened habitual action control in a non-food context. Consequently, although there is previous indication for obesity-associated domain-general behavioral inflexibility as suggested by paradigms assessing inhibitory control (Nederkoorn et al., 2006; Gunstad et al., 2007) or decision making (Pignatti et al., 2006; Weller et al., 2008; Horstmann et al., 2011; Simmank et al., 2015), these inflexibilities may not extend to abnormal stimulus-response linkage at the expense of outcome-driven action control. On the other hand, obesity-associated impairments in goal-directed behavior and overreliance on habits might not be generalizable, but may be restricted to the food domain. Previous investigations demonstrated food-related behavioral inflexibility in obesity as measured by food-specific versions of the delay-discounting task (Rasmussen et al., 2010) and the go/no-go task (Batterink et al., 2010; He et al., 2014). In the delay-discounting paradigm of Rasmussen et al. (2010) also a monetary condition was included. Interestingly, only food-specific discounting was significantly associated with the percentage of body fat. A recent study also showed a positive relationship between BMI and a behavioral estimate of habitual responding for snack food (Horstmann et al., 2015a). Unfortunately, the majority of these studies focused on the food context without including a comparative neutral condition. Therefore, conclusions regarding specificity of the effects should be drawn with caution. Moreover, abnormalities in habit formation might be a specific issue of certain obese subtypes as opposed to applying generally to obesity. Especially individuals with a diagnosis of binge eating (Voon et al., 2015; Reiter et al., 2016) or food addiction (Gearhardt et al., 2009; Davis et al., 2011; Davis, 2013; Meule and Gearhardt, 2014; Murphy et al., 2014) may be prone to stimulus-guided behavior, which should be clarified in future studies.

Impulsivity, or more specifically the domain of *Sensation Seeking*, negatively correlated with the DSI of both the slips-of-action and baseline test. In other words, *Sensation Seeking* was related to the inability to selectively respond for still-valuable outcomes and stimuli while suppressing responses for devalued outcomes and stimuli. This result suggests a general impairment in the ability to inhibit previously learnt responses. The finding of relationships between impulsivity and both balance measures (slips-of-action and baseline test) is consistent with the possibility of enhanced S→R learning and a relative reliance on habits in sensation seekers, but fits less well with the idea of impaired outcome-based learning (as the baseline test controls for outcome-based responding). On the other hand, the negative relationship of impulsivity and accuracy in the outcome-devaluation test does indicate a more specific dysregulation of outcome-based responding. Consequently, the current results are not sufficient to determine if the habit propensity in highly impulsive individuals is due to excessive reliance on habits or weak goal-directed control. An alternative interpretation of our results would be a general impairment in response inhibition in highly impulsive individuals leading to failures to suppress learnt responses across these different tests. Future studies should therefore control for this type of inhibition failure, to cancel out the possibility of inhibition disturbances which are independent of associative learning. Beyond the direct relation between task performance and impulsivity, we failed to find a moderation of the proposed relationship between BMI and the balance measure. Future studies focusing on the abovementioned subtypes of obesity may find interaction effects.

The finding of a habit propensity in impulsivity is in line with previous research showing impaired goal-directed control (Hogarth et al., 2012), cognitive inflexibility (Franken et al., 2008; Crews and Boettiger, 2009; Romer et al., 2009) and heightened cue-reactivity resulting in inhibition failures (reviewed in Bari and Robbins, 2013) in human impulsivity. Interestingly, in this study overreliance on habits was specifically observed in participants scoring high in the subdomain of *Sensation Seeking*, conceptualized as (1) the tendency to enjoy and pursue activities that are exciting and (2) the openness to trying new experiences that may be risky (Whiteside and Lynam, 2001). The abovementioned study of Hogarth et al. (2012) reported a negative relationship between goal-directedness and *Motor Impulsiveness* (Patton et al., 1995) in a food context. This conceptualization of impulsivity reflects the tendency to act on the spur of the moment, which probably covers aspects reflecting risk-taking and excitement seeking. Sensation seeking is assumed to go along with strong approach behaviors and weak behavioral avoidance/inhibition (Depue and Collons, 1999; Collins et al., 2012). In addition to that, sensation seeking or the related constructs of novelty or excitement seeking (Zuckerman, 1979; Cloninger et al., 1993) have been associated with the inability to delay responding in the face of a larger reward (Cyders and Coskunpinar, 2011), with risky and unpredictable decision making (Finn, 2002; Suhr and Tsanadis, 2007; Bornovalova et al., 2009; Noël et al., 2011), as well as with impairments in response inhibition (Finn, 2002; Noël et al., 2011). Another study indicates a diminished ability of sensation seekers to evaluate the negative outcomes in their decisions (Cservenka et al., 2013). According to our findings, these alterations may be due to heightened reliance on S→R guided action control with inappropriate consideration of outcome values. Individual differences in corticostriatal loops, previously associated with trait impulsivity, might constitute the underlying mechanism (Jentsch and Taylor, 1999; Volkow and Fowler, 2000; Cardinal et al., 2001; McClure et al., 2004; Brown et al., 2006; Dalley et al., 2008; Forbes et al., 2009; Davis et al., 2013), which should be clarified in future studies. Striatal dopaminergic disturbances in sensation seekers may contribute to these alterations (Golimbet et al., 2007; Munafò et al., 2008; Gjedde et al., 2010).

Apart from impulsivity cognitive capacities, as assessed by estimates of non-verbal IQ and short-term memory, were related to the balance between outcome- and stimulus-based responding. This is in line with previous research showing goal-directed behavior to depend on higher-order cognitive resources (Eppinger et al., 2013; Otto et al., 2013; Schad et al., 2014). According to their relationships with instrumental learning and performance, we recommend including estimates of IQ and working or short-term memory as standard control variables into studies investigating instrumental performance.

This study is accompanied by some limitations. Most substantial may be the issue of low task difficulty and corresponding ceiling effects. Therefore, the paradigm may not be sensitive enough to detect subtle differences in goal-directed vs. habitual behavior potentially associated with weight status. Future studies might adapt the task to make it more challenging, e.g., by consideration of the originally introduced congruent and incongruent discrimination conditions (de Wit et al., 2007). Thereby variance may be enahnced in order to disentangle potential subtle relationships. Furthermore, the instrumental learning phase may be adapted. To strengthen habit formation overtraining might be a fruitful approach (Tricomi et al., 2009). On the other hand, alternative tasks assessing behavioral flexibility, e.g., reversal learning tasks (e.g., Cools et al., 2006; Waltz et al., 2013; Zhang et al., 2014; Culbreth et al., 2015), might be more sensitive to detect subtle differences with respect to weight status. Moreover, domain-general impairments in goal-directed behavior may play a role in gaining weight and in the end obesity if food motivation is also strong (Nederkoorn et al., 2010; Rollins et al., 2010; Appelhans et al., 2011). Unfortunately, with the current paradigm we cannot evaluate the aspect of food motivation, which should be considered in future investigations. Apart from limitations of the paradigm, sample characteristics might have limited the ability to detect differences. Unfortunately, we were not able to do analyses based on the aspect of food addiction, as only few participants showed symptoms and just seven participants were diagnosed as food addicted (Gearhardt et al., 2009). In addition, here we focused on young and healthy individuals. Impairments in goal-directed behavior might be specifically apparent in morbidly obese (Horstmann et al., 2015b) or older obese individuals (de Wit et al., 2014). Future studies therefore may broaden age and BMI range. Moreover, with the current paradigm we cannot draw conclusions on the generalizability of the effect. Future comparative studies, using paradigms that differentiate between neutral and specific (e.g., food) contexts, are needed to clarify this aspect. Finally, according to low task difficulty one may argue that differences in participants’ motivation might have accounted for the effect of *Sensation Seeking*. Although we cannot rule out this possibility, providing participants with monetary reward – a universally effective reinforcer – is expected to maintain high motivation in all participants, especially as there is no previous indication for differences in the sensitivity to reward depending on *Sensation Seeking*. Also the excellent - near ceiling - performance among all participants indicate continuous engagement in the task.

## Conclusion

This study is indicative of an impaired balance between goal-directed and habitual action control in the impulsivity domain of *Sensation Seeking* leading to a habit propensity. However, we failed to find an imbalance between goal-directed and habitual behavior in overweight or obesity in a non-food context. We recommend that future studies should (1) adapt the applied task to make it more challenging, thereby enhancing variance in task performance, and (2) focus on specific subtypes of obesity which might be especially prone to an overreliance on habits. Moreover, neuroimaging may contribute to a more detailed specification of proposed habit propensities by identifying brain structures mediating differences in action control.

## Author Contributions

AD, AH, and SdW are responsible for study conception and design. AD acquired the data and did the analysis. AD, AH, and SdW interpreted the results. AD did the drafting of the work. AD, AH, and SdeW revised the draft critically, approved the final version for publication and agreed to be accountable for all aspects of the work, ensuring that questions related to the accuracy or integrity of any part of the work are appropriately investigated and resolved.

## Conflict of Interest Statement

The authors declare that the research was conducted in the absence of any commercial or financial relationships that could be construed as a potential conflict of interest.

The reviewer MP and handling Editor declared their shared affiliation, and the handling Editor states that the process nevertheless met the standards of a fair and objective review.
