# Supplementary material for: General Habit Propensity Relates to the Sensation Seeking Subdomain of Impulsivity But Not Obesity
Source: Front Behav Neurosci. 2016 Nov 8;10:213. doi: 10.3389/fnbeh.2016.00213 (PMC5099246; doi:10.3389/fnbeh.2016.00213)
Supplement: Supplementary file 1 [file Data_Sheet_1.PDF]

Table SI. Spearman correlations between variables.

		VPA score	IQ	Sensation Seeking	Perseverance	Premeditation	Urgency	Age	YFAS symp
**BMI (kg/m^2)**	ρ	.001	-.048	.001	.064	.017	.040	.013	.372
	p	.992	.629	.992	.519	.865	.684	.897	<.001
**YFAS symp**	ρ	.082	-.110	-.211	-.016	-.190	.350	-.187	
	p	.408	.266	.030	.875	.053	<.001	.056	
**Age**	ρ	-.099	-.203	-.003	.014	.013	-.132		
	p	.314	.038	.975	.886	.895	.180		
**Urgency**	ρ	-.121	-.023	-.054	.344	.113			
	p	.222	.817	.583	<.001	.253			
**Premeditation**	ρ	-.192	-.007	.313	.149				
	p	.050	.947	.001	.128				
**Perseverance**	ρ	-.027	-.074	-.160					
	p	.781	.456	.102					
**Sensation Seeking**	ρ	-.134	-.153						
	p	.172	.119						
**IQ**	ρ	.293							
	p	.002							

Table SII. Normality tests on outcome variables (Kolmogorov-Smirnov test).

	D	df	p
**outcome-devaluation test**			
accuracy (%)	.274	105	<.001
**slips-of-action test**			
DSI	.178	105	<.001
**baseline test**			
DSI	.192	105	<.001
**instrumental learning phase**			
accuracy (%) block 1	.130	105	.004
accuracy (%) block 2	.229	105	<.001
accuracy (%) block 3	.259	105	<.001
accuracy (%) block 4	.292	105	<.001
accuracy (%) block 5	.372	105	<.001
accuracy (%) block 6	.329	105	<.001
accuracy (%) block 7	.432	105	<.001
accuracy (%) block 8	.441	105	<.001

DSI devaluation sensitivity score

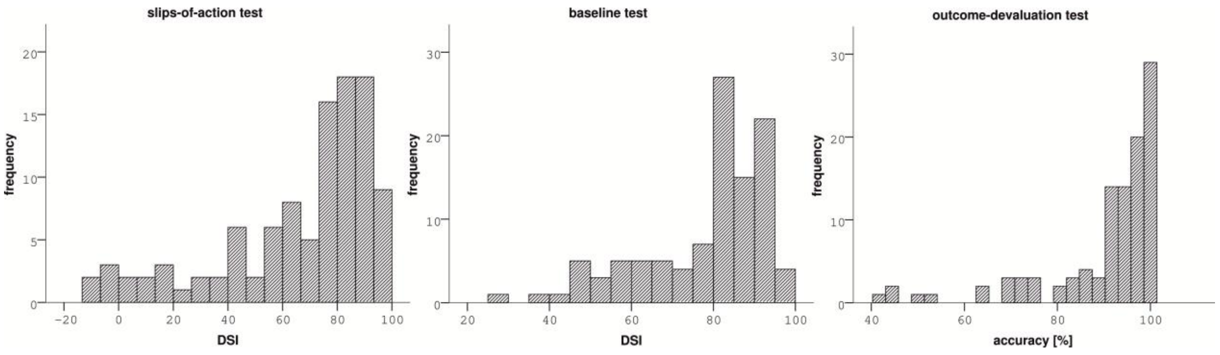

Figure SI. Distribution of measures of the slips-of-action, baseline, and outcome-devaluation tests. Abbreviations: DSI devaluation sensitivity index.

Table SIII. Reaction times (RT) in the slips-of-action, baseline, and outcome-devaluation tests (1st Quartile, Median, 2nd Quartile). Included are RT of responses for devalued and still-valuable outcomes (slips-of-action and baseline tests) and RT of responses on the outcome-devaluation test.

	**slips-of-action test**		**baseline test**		**outcome-devaluation test**
interquartile range	devalued (ms)	valuable (ms)	devalued (ms)	valuable (ms)	(ms)
**1st Quartile**	609.25	650.95	594.00	609.00	750.25
**Median**	672.00	698.32	656.00	649.00	867.50
**2nd Quartile**	736.50	736.86	742.00	695.00	1083.75

slips-of-action test: no responses for devalued outcomes n=4, **baseline test:** no responses for devalued stimuli n=5

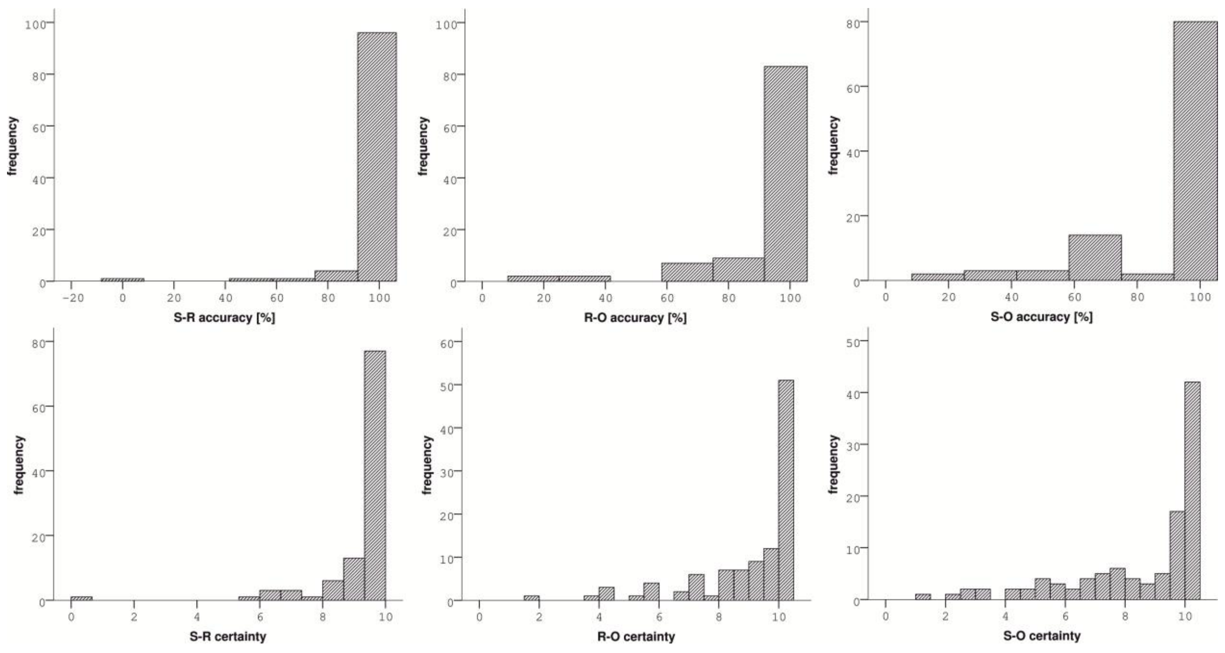

Figure SII. Distribution of accuracy [%] and certainty on S→R, R→O, and S→O associations as indicated in paper-and-pencil questionnaires on contingency knowledge.
